# Development and application of a *Puccinia triticina* avirulence gene *AvrLr15*-specific molecular marker

**DOI:** 10.3389/fpls.2025.1668725

**Published:** 2025-10-08

**Authors:** Yuqing Jin, Zhongchi Cui, Maoxing Song, Shitao Yuan, Dashuo Ding, Yuanxia Liu, Shuxin Yin, Peiyuan Xu, Chengyi Yan, Daqun Liu, Zhihui Wu, Haiyan Wang

**Affiliations:** ^1^ College of Plant Protection, Hebei Agricultural University, Technological Innovation Center for Biological Control of Crop Diseases and Insect Pests of Hebei Province, Baoding, Hebei, China; ^2^ Wheat Research Institute, Tangshan Academy of Agricultural Sciences, Tangshan, Hebei, China; ^3^ National Engineering Research Center for Agriculture in Northern Mountainous Areas, Hebei Agricultural University, Baoding, China

**Keywords:** *Puccinia triticina*, avirulence gene, molecular marker, virulence monitoring, AvrLr15

## Abstract

*Puccinia triticina* (*Pt*) races vary frequently, and new virulence races continue to emerge, leading to a lack of durable resistance in wheat cultivars. Therefore, monitoring avirulence gene composition and variation in *Pt* is crucial. In our previous study, we identified an avirulence protein AvrLr15 from *Pt* that induced *Lr15*-dependent immune responses and found that evasion of *Lr15*-mediated resistance in wheat was associated with a deletion and point mutations of amino acids in AvrLr15. In this study, TcLr15–Thatcher hybrid materials and *Lr15* mutant materials were developed, which further confirmed the reliability of *AvrLr15*. A duplex PCR assay using two primer pairs (M1-F/M1-R and M2-F/M2-R) designed based on the differences between *AvrLr15* and virulence gene *avrLr15* successfully differentiated avirulent and virulent races, which can be used as the *AvrLr15* marker. A total of 21 races with different virulence to *Lr15* were tested to confirm the reliability of the *AvrLr15* molecular marker. A total of 168 *Pt* isolates were collected from 15 provinces in 2024, and the *AvrLr15* molecular marker can monitor the distribution of the V15 (virulent to the *Lr15* gene) race and assess the durability of *Lr15*-mediated resistance effectively. Our findings developed the first *Pt* avirulence gene molecular marker to monitor natural *Pt* populations and guide the deployment of *Lr15*-resistant wheat cultivars in the field.

## 1 Introduction

Wheat leaf rust, caused by *Puccinia triticina* (*Pt*), is one of the most economically significant fungal diseases worldwide, leading to substantial yield losses in major wheat-producing regions (Eversmeyer and Kramer, 2000). The widespread cultivation of single resistant varieties exerts selective pressure on *Pt* populations, resulting in the emergence of new virulent races and shifts in dominant pathotypes. This dynamic leads to the breakdown of host resistance and subsequent disease epidemics, causing severe production losses (Aime et al., 2018; Ellis et al., 2014).

To mitigate the rapid erosion of resistance in *Pt*-resistant cultivars, continuous monitoring of *Pt* virulence in field populations is essential. To date, over 100 leaf rust resistance (*Lr*) genes have been identified in wheat and its wild relatives, with 85 formally designated (Sharma et al., 2024). The *Lr15* gene, derived from common wheat (*Triticum aestivum*) cultivar Kenya and located on chromosome 2D, confers resistance to *Pt* races in Canada, Europe, and Central Asia. However, its effectiveness has declined in Germany, India, and South Africa (Huerta-Espino et al., 2011). Statistical analysis of the *Pt* physiological races in China’s major wheat-producing regions from 2007 to 2021 revealed that virulence frequency to *Lr15* presented a general trend of fluctuation and decline (Li et al., 2024; Zhang et al., 2020). In addition, *Lr15* is present in only a few commercial cultivars, indicating its limited deployment in field production. This low utilization indicates that *Lr15* has application potential (Gao et al., 2019) in the future. Thus, developing effective management strategies to preserve *Lr15* effectiveness and prevent large-scale epidemics is critical.

Gene-for-gene theory posits that effective resistance occurs only when a plant carries a functional *R* gene and the corresponding pathogen carries an *Avr* gene. In contrast, loss-of-function variations in *Avr* genes (deletions, point mutations) or *Avr* gene absence disrupts this recognition, allowing the pathogen to evade *R*-mediated resistance (Flor, 1971; Ellis et al., 2014). Molecular markers of the *Avr* gene are essential tools for detecting polymorphisms in *Avr* genes during pathogen surveillance (Panthee, 2023), as they enable rapid detection of pathogen avirulent/virulent races and support dynamic monitoring of field virulence. Single-nucleotide polymorphism (SNP) markers offer particular advantages due to their high stability, rapid detection, and suitability for high-throughput analysis (Terracciano et al., 2013; Al-Samarai and Al-Kazaz, 2015).

In our previous study, we identified the *Pt* effector Pt_19 as the avirulence gene *AvrLr15* and characterized four nucleotide mutations in its coding sequence that differentiate the avirulent (*AvrLr15*) and virulent (*avrLr15*) alleles (Cui et al., 2024). Here, we further validated the function of *AvrLr15* by generating TcLr15–Thatcher hybrids and EMS-induced TcLr15 mutant lines and developed an *AvrLr15*-specific molecular marker and applied it to systematically monitor the distribution of the V15 (virulent to the *Lr15* gene) races across China’s major wheat-growing regions. Our findings support strategic deployment of *Lr15*-based wheat cultivars to improve durable resistance against *Pt* epidemics.

## 2 Materials and methods

### 2.1 Plant materials, *Pt* isolates, and sample collection

Wheat near-isogenic line TcLr15 (carrying the *Lr15* gene in the Thatcher background) and its susceptible parent Thatcher, along with 21 *Pt* isolates with different virulence to TcLr15 from different years, were maintained at the Wheat Leaf Rust Laboratory of Hebei Agricultural University (**Supplementary Table S1**). A total of 168 *Pt* single-pustule isolates were generated in the greenhouse from field-collected leaves in 2024 (**Supplementary Table S2**). Each isolate was systematically numbered according to its geographic origin.

### 2.2 Generation and characterization of TcLr15–Thatcher hybrid materials

TcLr15 and Thatcher were hybridized to obtain the F_1_ generation (Li et al., 1992), which was then selfed to obtain the F_2_ generation of TcLr15–Thatcher hybrid material. The *Lr15* molecular marker was used to detect *Lr15* in TcLr15–Thatcher hybrid materials (F_2_ generation). TcLr15, Thatcher, and the F_2_ generation of TcLr15–Thatcher hybrid material were infiltrated with pure protein of AvrLr15, respectively, and the programmed cell death (PCD) on the infiltrated region was observed at 24 h. The test was repeated three to five times.

### 2.3 EMS mutagenesis and mutant screening

To obtain wheat materials with *Lr15* gene mutation, mutagenesis was performed as previously described (Wang et al., 2023). In brief, 200 TcLr15 seeds were soaked in 100 mL of 0.4% EMS, mixed by 125 r min^−1^ shaking at 25 °C for 10 h, and washed with running water at 26°C for 4 h. Treated seeds were planted in a greenhouse maintained at 25 °C for 15 days to observe seedling growth and development. Putative mutants were screened using *Lr15*-specific molecular markers and avirulent *Pt* race PHNT inoculation.

### 2.4 Design and validation of the avirulence gene *AvrLr15* molecular marker

Based on the four nucleotide variation sites previously identified between the *avrLr15* and *AvrLr15* sequences, a total of 30 primer pairs were designed targeting the CDS region to serve as molecular markers for *AvrLr15* (**Supplementary Table S3**). The primers were designed using Primer 5.0 and subsequently synthesized by Sangon Biotech (Shanghai) Co., Ltd. Genomic DNA was extracted from urediniospores of 21 *Pt* races that exhibited different infection types on TcLr15, using a modified CTAB method based on the protocol by Doyle (Doyle and Doyle, 1987). PCR amplification was performed using specific primers in a total reaction volume of 25 μL, containing 12.5 μL of 1× Taq Master Mix, 1 μL each of 5 mmol/L forward and reverse primers, and 1 μL of 30-ng DNA template, with the remaining volume supplemented with ddH_2_O. The cycling conditions are provided in **Supplementary Table S4** (the reaction system and program for the *β-Actin* internal reference were identical). The presence of an amplification product of the expected size was indicative of an avirulent isolate, whereas its absence indicated a virulent isolate.

### 2.5 Application of the avirulence gene *AvrLr15* molecular marker

In 2024, a total of 168 *Pt* isolates were collected from 15 provinces across China, with Hebei and Henan provinces exhibiting relatively higher sampling densities compared with other regions. Samples were collected from three sites per province where natural infections had occurred and no fungicides had been applied. At each site, three biological replications were conducted within 10 m × 10 m experimental plots using the five-point sampling method for isolate collection (Saghai-Maroof et al., 1984). Following collection, *Pt* isolates were inoculated onto TcLr15 and Thatcher wheat cultivars, with three biological replicates per isolate, for virulence phenotyping under controlled environmental conditions (22 ± 2 °C, 70% relative humidity, 150–250 μmol·m^−2^·s^−1^ light intensity, and 12 h light/12 h dark). Genomic DNA was extracted from each isolate, and virulence was confirmed through PCR amplification using specific molecular markers (M1 and M2) (**Supplementary Table S3**). Statistical analysis was performed on the spatial distribution of V15 virulence frequency across different geographical regions. Chi-square tests were applied to the virulence frequency results, wherein the likelihood ratio method was adopted when the sample size (N) exceeded 40 and the minimum expected count fell between 1 and 5.

## 3 Results

### 3.1 The specific recognition between *AvrLr15* and *Lr15* was confirmed in hybrid plants

To provide the genetic proofs for the specific interaction between the avirulence gene *AvrLr15* and the resistance gene *Lr15*, F_2_ progeny derived from the TcLr15–Thatcher hybrid were genotyped using an *Lr15*-specific molecular marker. Among 23 F_2_ hybrid plants, a fragment of 1,000-bp length was detected in 14 plants, which was identical to the TcLr15 as positive control, whereas nine plants exhibited a 500-bp fragment, matching Thatcher as negative control (**Supplementary Figure S1**), confirming the presence of *Lr15* in 14 F_2_ hybrid plants. Chi-square test confirmed that the 14:9 segregation ratio matches the expected 3:1 mendelian ratio (χ^2^ = 2.43, df=1, P>0.05), supporting *Lr15* as a single dominant gene.

**Figure 1:**
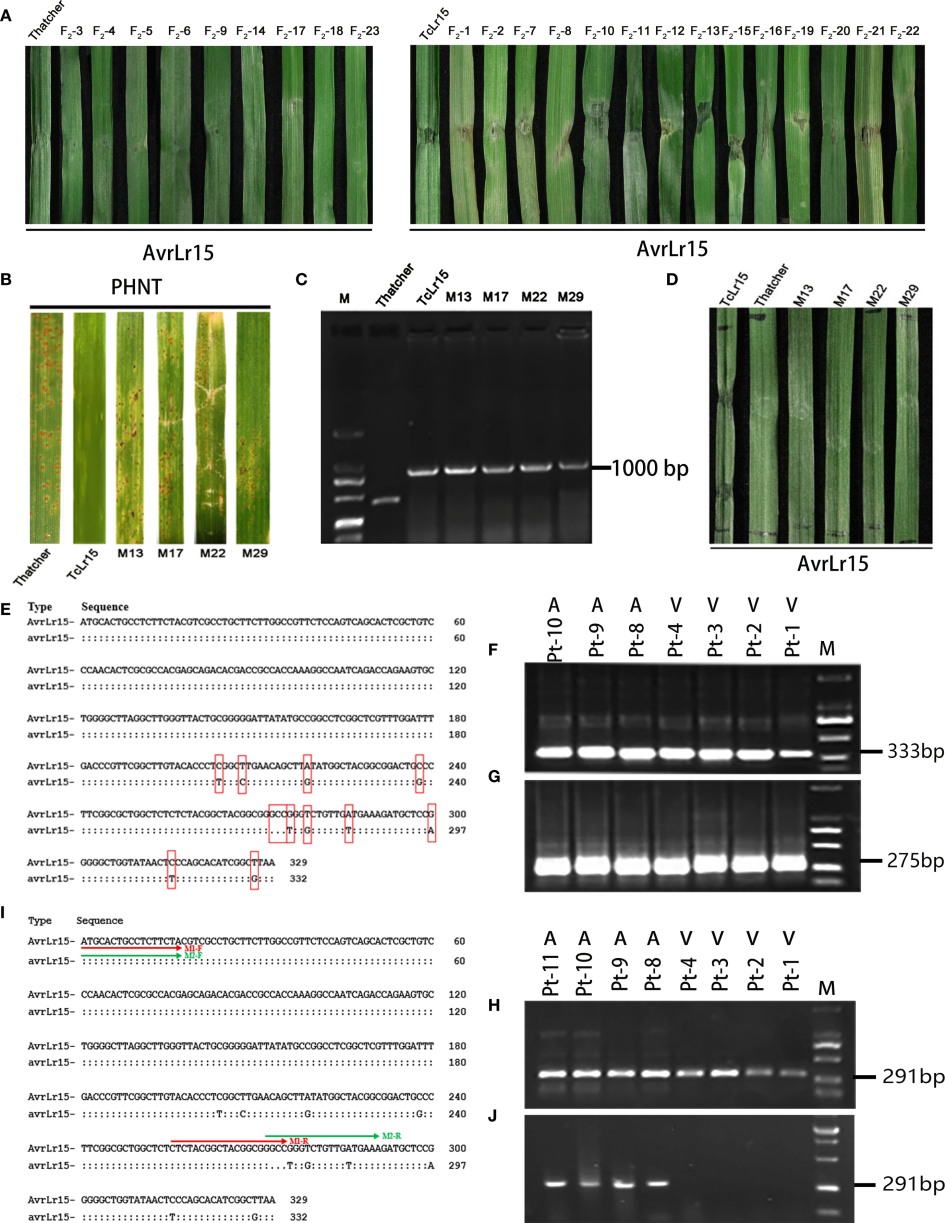
Validation of the avirulence gene AvrLr15 in *Pt* and molecular marker development. **(A)** Transient expression analysis of AvrLr15 in the hybrid wheat of TcLr15 and Thatcher, F_2_-1-F_2_-23: second hybrid generation of TcLr15–Thatcher. **(B)** Inoculation of TcLr15 mutants with the avirulent strain PHNT (against *Lr15*). **(C)** Detection of TcLr15 mutants using molecular markers for the *Lr15* gene. **(D)** Transient expression analysis of AvrLr15 in TcLr15 mutants. **(E)** Schematic diagram of nucleotide sequence polymorphisms between the virulence (*avrLr15*) and avirulence (*AvrLr15*) alleles. **(F)** PCR amplification using primer M17 (333 bp) on four virulent and three avirulent races. **(G)** PCR amplification using primer M1 (275 bp) on four virulent and three avirulent races. **(H)** PCR amplification using primer M2 (291 bp) on four virulent and four avirulent races. **(I)** Primer positions are indicated by colored lines: M1 (red) and M2 (green). **(J)** Dual PCR amplification using primers M1 and M2, followed by electrophoresis, on four virulent and four avirulent races.

To verify whether *AvrLr15* could trigger an immune response in the TcLr15–Thatcher hybrid wheat, we infiltrated purified AvrLr15 protein into the F_2_ hybrid materials. Nine *Lr15*-negative hybrid plants did not display PCD response, consistent with the negative control (Thatcher infiltrated with *AvrLr15*). In contrast, 14 *Lr15*-positive hybrid plants developed PCD response upon AvrLr15 infiltration, identical to the positive control (TcLr15 infiltrated with *AvrLr15*) (**Figure 1A**). These results demonstrate that AvrLr15 can specifically induce an immune response in *Lr15*-containing TcLr15–Thatcher hybrid materials, providing genetic evidence for the specific recognition between *AvrLr15* and *Lr15*.

### 3.2 *AvrLr15* could not trigger the immune response in the *Lr15* mutants

Based on statistical analysis of germination rate and malformation frequency in TcLr15 wheat seeds treated with varying EMS concentrations (**Supplementary Table S5**), 0.4% EMS was identified as the optimal concentration for subsequent mutagenesis treatments. Phenotype assessments showed that four M_0_ plants (M13, M17, M22, and M29) displayed an intermediate susceptible phenotype (“3” infection type), whereas TcLr15 exhibited an immune response (“0” infection type), and Thatcher was highly susceptible (“4” infection type) (**Figure 1B**). Further PCR amplification using an *Lr15*-specific molecular marker confirmed that four M_0_ mutants retained the *Lr15* gene, as evidenced by a 1,000-bp amplification product (**Figure 1C**). These results indicate that the *Lr15* gene harbored mutations in the four mutant lines, M13, M17, M22, and M29.

To determine whether mutations in *Lr15* affect its recognition with *AvrLr15*, purified AvrLr15 protein was infiltrated into the TcLr15 mutants. As expected, TcLr15 plants as positive control developed a PCD response, whereas four mutants (M13, M17, M22, and M29) did not, similar to the susceptible Thatcher as negative control (**Figure 1D**). These results demonstrate that mutations in *Lr15* disrupt its recognition with *AvrLr15*.

### 3.3 Development of a molecular marker for avirulence gene *AvrLr15*

To develop molecular markers for *AvrLr15*, allele-specific primers were designed based on the 11 nucleotide variations identified between the *AvrLr15* and *avrLr15* alleles, including 10 single-nucleotide polymorphisms (SNPs: 204_C→T_, 208_T→C_, 219_A→G_, 238_C→G_, 276_G→T_, 278_T→G_, 286_A→T_, 300_G→A_, 316_C→T_, and 326_T→G_) and one trinucleotide deletion (273_GCC→-_) (**Figure 1E**). A total of 30 primer pairs were synthesized and initially screened using genomic DNA from seven to eight *Pt* races with defined virulence profiles. However, similar amplification products were observed between avirulence and virulence races to *Lr15* (**Figures 1F-H**), suggesting that single SNP primer pairs could not distinguish the polymorphism between avirulence and virulence races to *Lr15*.

Subsequently, duplex PCR assays were performed using upstream primers (M1-F and M2-F) designed from the first 21 nucleotides of *AvrLr15*/*avrLr15*, coupled with deletion-flanking reverse primers: M1-R (3'-terminus anchored to the GCC deletion site) and M2-R (5'-terminus initiated at the deletion site) (**Figure 1I**). A 291-bp fragment exclusively was obtained in avirulent races to *Lr15*, whereas no amplification product appeared in the virulent races to *Lr15* (**Figure 1J**). These results demonstrated the potential of M1-F/M1-R and M2-F/M2-R primer pairs for discriminating avirulent and virulent races to *Lr15*.

### 3.4 Validation of the *AvrLr15*-specific molecular marker

**Figure 2:**
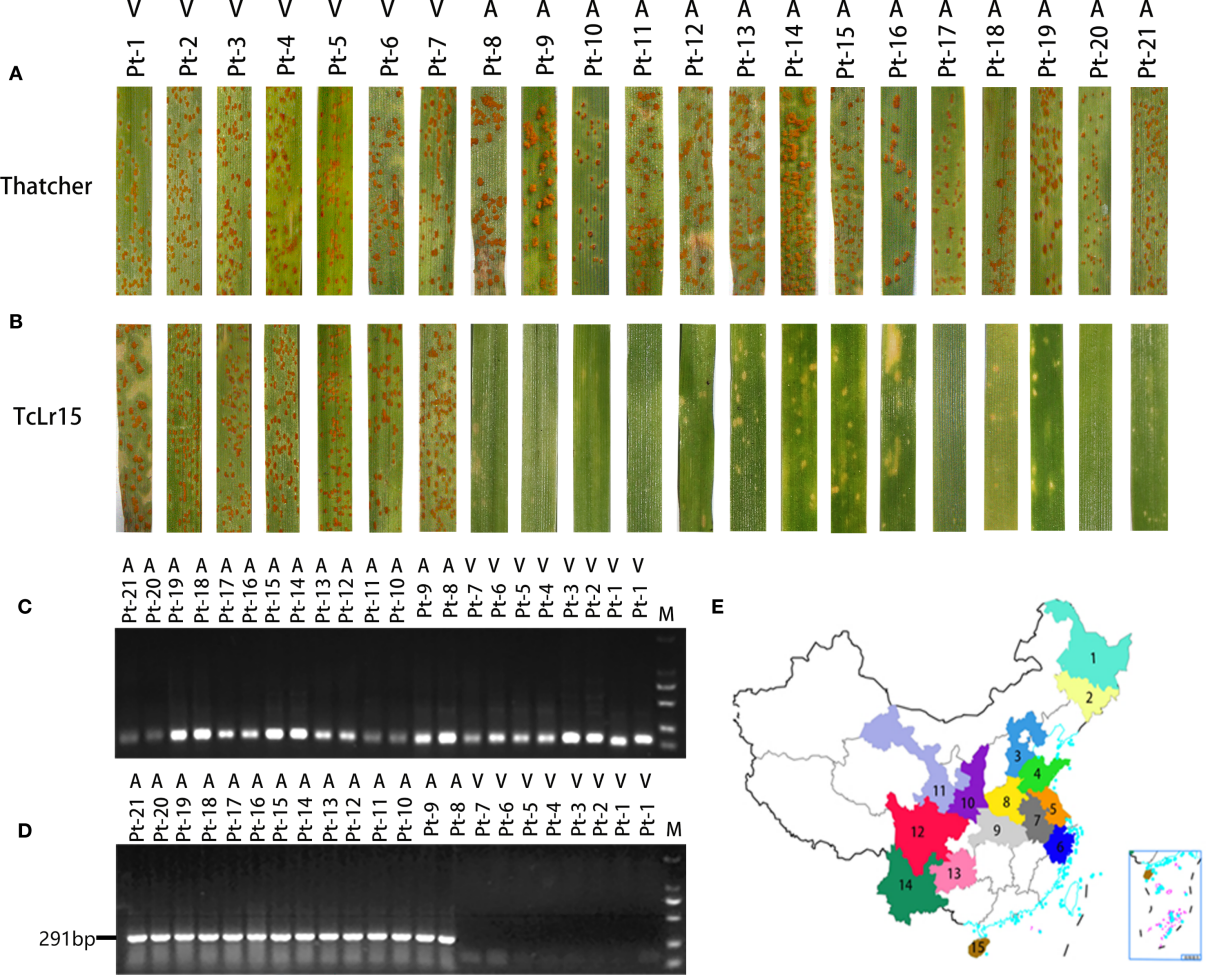
Validation and application of molecular markers for the wheat avirulence gene *AvrLr15*. **(A)** Phenotypic identification of 21 *Pt* physiological races inoculated on the susceptible material Thatcher. **(B)** Phenotypic identification of 21 *Pt* physiological races inoculated on TcLr15 wheat. **(C)** PCR detection of the *β-actin* reference gene in 21 *Pt* physiological races. **(D)** Dual PCR amplification using primers M1 and M2, followed by electrophoresis, on DNA from 21 *Pt* physiological races. **(E)** Sampling regions for 2024 *Pt* isolates. Colored areas indicate collection sites: 1: HLJ (Heilongjiang); 2: JL (Jilin); 3: HEB (Hebei); 4: SD (Shandong); 5: JS (Jiangsu); 6: ZJ (Zhejiang); 7: AH (Anhui); 8: HEN (Henan); 9: HUB (Hubei); 10: SX (Shanxi); 11: GS (Gansu); 12: SC (Sichuan); 13: GZ (Guizhou); 14: YN (Yunnan); 15: HAN (Hainan).

To elucidate sequence variations in *AvrLr15* among different virulent races of *Pt*, we systematically assessed virulence profiles against *Lr15*. Physiological races Pt-1, Pt-2, Pt-3, Pt-4, Pt-5, Pt-6, and Pt-7 exhibited virulence toward TcLr15, developing abundant urediniospores with limited necrotic lesions—resembling disease susceptibility in Thatcher as controls. Conversely, races Pt-8, Pt-9, Pt-10, Pt-11, Pt-12, Pt-13, Pt-14, Pt-15, Pt-16, Pt-17, Pt-18, Pt-19, Pt-20, and Pt-21 showed avirulence to *Lr15*, inducing extensive necrotic responses without urediniospore formation on TcLr15 (**Figures 2A, B**; **Supplementary Table S1**). Collectively, 7 virulent races (Pt-1, Pt-2, Pt-3, Pt-4, Pt-5, Pt-6, Pt-7) and 14 avirulent races (Pt-8, Pt-9, Pt-10, Pt-11, Pt-12, Pt-13, Pt-14, Pt-15, Pt-16, Pt-17, Pt-18, Pt-19, Pt-20, Pt-21) were identified.

To further validate the reliability of the *AvrLr15* molecular marker in distinguishing virulent and avirulent races, PCR amplification was conducted using genomic DNA from 21 characterized *Pt* races. Initial quality control was performed through *β-actin* gene amplification, confirming the integrity of all DNA templates (**Figure 2C**). Subsequent electrophoresis analysis revealed distinct patterns, a 291-bp amplification band appeared in 14 avirulent races, whereas 7 virulent races exhibited no amplification (**Figure 2D**). These results demonstrate that the *AvrLr15* molecular marker can accurately differentiate *Pt* races with different virulence to *Lr15*.

### 3.5 Application of the *AvrLr15* molecular marker

To monitor the distribution of the V15 race and assess the durability of *Lr15*-mediated resistance, 168 *Pt* isolates were collected from 15 provinces in 2024, predominantly in the Huang-Huai-Hai wheat region (**Figure 2E**; **Supplementary Table S2**). Phenotypic assessment after inoculation on TcLr15 classified 120 isolates as avirulent (immune response) and 48 isolates as virulent (highly susceptible). DNA quality verification followed by *AvrLr15*-specific marker testing confirmed that the 291-bp band was tested in 120 avirulent isolates, whereas the 291-bp band was absent in 48 virulent isolates (**Supplementary Figure S2A, B**). *Lr15* virulent isolates were distributed as follows: Hebei (14), Henan (9), Shandong (6), Hubei (5), Zhejiang (5), Sichuan (4), Gansu (3), and Shanxi (2).

Statistical analysis of virulence frequency revealed that the V15 virulent strain was distributed at relatively high frequencies in Hubei (45.45%), Hebei (43.75%), Henan (39.13%), and Zhejiang (38.46%), whereas lower frequencies were observed in Gansu (37.50%) Shandong (35.29%), Shanxi (28.57%), and Sichuan (26.67%). The V15 isolates were not detected in any sampled isolates from Yunan, Jiangsu, Anhui, Heilongjiang, Hainan, Liaoning, and Guizhou. The chi-square test revealed significant differences in the V15 virulence frequency among regions (χ^2^ = 19.234, P = 0.007<0.05). Specifically, the Huang-Huai area exhibits significant differences in the V15 virulence frequency compared with the southwestern/northeastern provinces (**Supplementary Table S6**), supporting that V15 virulent strains show significant geographical different in China’s wheat regions.

## 4 Discussion

Plant resistance is generally conferred by immune receptors of the nucleotide-binding leucine-rich repeat (NLR) class, which recognize pathogen effector proteins delivered into the host cell during infection, often known as avirulence proteins. *Phytophthora infestans* avirulence genes *Avramr1* was identified and verified by transiently co-expressed with potato late blight resistance gene *Rpi-amr1* in *Nicotiana benthamiana* (Lin et al., 2020). Lin et al. (2022) identified *P. infestans* avirulence gene *Avramr3* that is recognized by potato late blight *Rpi-amr3*. Wheat powdery mildew resistance gene *Pm1a* as an immune receptor gene specifically recognizes *Blumeria graminis* avirulence gene *AvrPm1a* (Hewitt et al., 2021). Müller et al. (2022) identified *Blumeria graminis* avirulence gene *AvrPm17* by transient co-expression in *Nicotiana benthamiana*. However, the genome of *Pt* is relatively complex and dikaryotic, which has resulted in significantly lagging research on its avirulence genes in this field. Our previous study identified *Pt* avirulence genes *AvrLr21* and *AvrLr15* (Shen et al., 2024; Cui et al., 2024), finding that *Lr21*-breaking *Pt* isolates can suppress *Lr21*-mediated immunity. To provide strong genetic evidence for the specific recognition between *AvrLr15* and *Lr15*, the hybrid plants of Thatcher and TcLr15 and *Lr15* EMS mutants were constructed in this study, and the function of *AvrLr15* as an avirulence gene was further verified.

The development of molecular markers for avirulence genes enables precise detection of gene variations, offering molecular evidence for understanding how pathogens evade host resistance recognition through genetic mutations (Dong et al., 2009). Tong et al. (2023) developed *Sw-5b*-specific markers to distinguish between *Sw-5bR* and *Sw-5bS* alleles, which enable precise selection and accelerating tomato spotted wilt virus (TSWV)-resistant tomato breeding to mitigate yield losses. *Phytophthora sojae* avirulence gene *Avr3a* exhibits two variation patterns, namely, coding sequence mutations and promoter-mediated transcriptional loss, facilitating the creation of two SNP markers and one PCR marker for virulence identification (Hu et al., 2021). Dussault-Benoit et al. (2020) developed a molecular assay method to define the pathotypes of *Phytophthora sojae*, based on seven *Avr* genes (*Avr1a*, *Avr1b*, *Avr1c*, *Avr1d*, *Avr1k*, *Avr3a*, and *Avr6*). The matching rate between the molecular assay method and the phenotyping assay was as high as 97%. Our previous study demonstrated that *Lr15*-breaking *Pt* isolates contained nucleotide deletions that led to one amino acid deletion located at amino acid residue P92 and multiple nucleotide changes that resulted in three amino acid substitutions at residues P80, M96, and P106, which provides a basis for the development of the *AvrLr15* molecular marker. We tried to distinguish the avirulence and virulence races to *Lr15* by 30 SNP-based primers, but no polymorphism was detected between the avirulence and virulence races to *Lr15* (**Figures 1F, G**). SNP-based primer designs rely on single-base variation. However, such variation can be easily masked by non-specific primer binding during PCR (Zhang et al., 2023). This limitation was obvious in our study: All 30 SNP primers failed to differentiate *AvrLr15* and *avrLr15* alleles, resulting in cross-amplification in both virulent and avirulent *Pt* races. A duplex PCR assay enables the effective differentiate of the alleles in a single PCR reaction (Ma et al., 2003). In this study, the molecular marker for *AvrLr15* was developed by duplex PCR assays and used to monitor the distribution of the V15 race. The virulence frequency of V15 was revealed in 168 *Pt* isolates collected from 15 provinces in China in 2024. The matching rate between the molecular assay and the phenotyping assay was as high as 100%. All these findings benefit the assess of the durability of *Lr15*-mediated resistance.

The *AvrLr15* molecular marker was used to monitor the distribution of the V15 race collected from 15 provinces in 2024; ongoing monitoring is needed to track temporal shifts. All in all, the molecular marker of *AvrLr15* as the first molecular marker for an avirulence gene of *Pt* was developed successfully, and it can rapidly detect *Pt* isolates with different virulence to *Lr15*. Our findings will provide a molecular tool for monitoring virulence in natural *Pt* populations and guiding the field deployment of *Lr15*-resistant wheat cultivars and contribute to maintaining the durable resistance endowed by wheat leaf rust resistance gene *Lr15*.

## Data Availability

The original contributions presented in the study are included in the article/**Supplementary Material**. Further inquiries can be directed to the corresponding authors.
