# Supplementary material for: Development and application of a Puccinia triticina avirulence gene AvrLr15-specific molecular marker
Source: Front Plant Sci. 2025 Oct 8;16:1668725. doi: 10.3389/fpls.2025.1668725 (PMC12540341; doi:10.3389/fpls.2025.1668725)
Supplement: Supplementary file 1 [file Supplementaryfile1.docx]

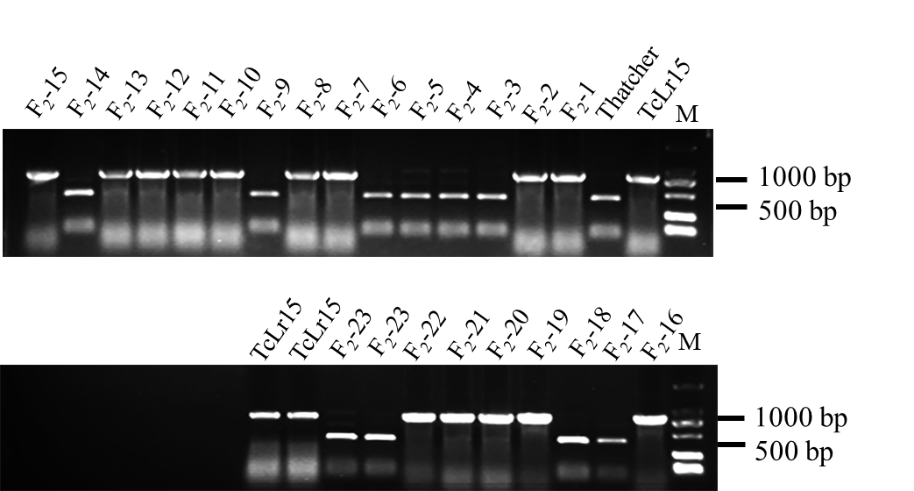


**Supplementary Figure S1. Detection of TcLr15-Thatcher hybrid materials.**

F_2_-1–F_2_-23: 23 F_2_ generation heterozygous plants.

**Supplementary Figure S2. Application of molecular markers for the avirulence gene *AvrLr15*.**

(A) PCR detection of the *β-actin* reference gene in 168 *Pt* isolates collected in 2024. Samples 1–32: Hebei; 33–49: Shandong; 50–72: Henan; 73–83: Hubei; 84–96: Zhejiang; 97–104: Gansu; 105–119: Sichuan; 120–126: Shanxi; 127–140: Jiangsu; 141–149: Yunnan; 150–159: Anhui; 160–162: Heilongjiang; 163–164: Hainan; 165–166: Liaoning; 167–168: Guizhou. (B) PCR detection of the *AvrLr15* molecular marker in the 2024 *Pt* isolates (same numbering as in panel A).


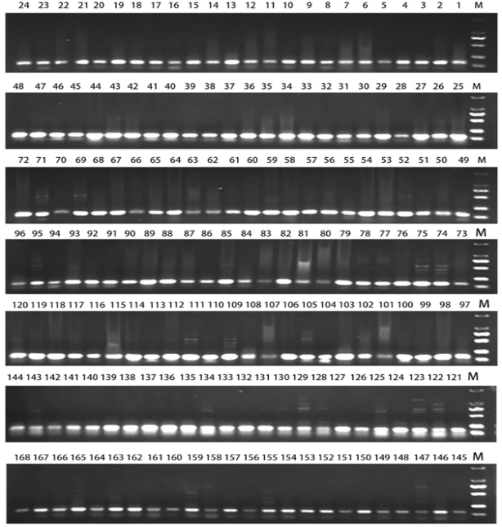


A


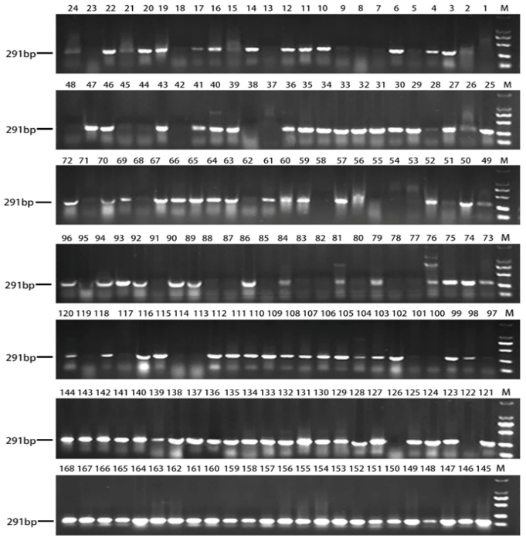


B
