# Supplementary material for: Development and application of a Puccinia triticina avirulence gene AvrLr15-specific molecular marker
Source: Front Plant Sci. 2025 Oct 8;16:1668725. doi: 10.3389/fpls.2025.1668725 (PMC12540341; doi:10.3389/fpls.2025.1668725)
Supplement: Supplementary file 2 [file Supplementaryfile2.docx]

**Supplementary Table S1. Statistics of existing physiological races of *Pt* and their virulence information against the *Lr15* gene.**

| Isolate | Year | Sampling  location | Virulence  test | Pathotype | |
| --- | --- | --- | --- | --- | --- |
|  |  |  |  | Thatcher | TcLr15 |
| Pt-1 | 2020 | Hebei, Chian | V | 3+ | 3+ |
| Pt-2 | 2021 | Henan, Chian | V | 4 | 3+ |
| Pt-3 | 2020 | Hebei, Chian | V | 4 | 4 |
| Pt-4 | 2022 | Shandong, Chian | V | 4 | 3+ |
| Pt-5 | 2023 | Hebei, Chian | V | 4 | 4 |
| Pt-6 | 2023 | Shandong, Chian | V | 4 | 4 |
| Pt-7 | 2023 | Hebei, Chian | V | 4 | 4 |
| Pt-8 | 2021 | Hebei, Chian | A | 4 | ； |
| Pt-9 | 2020 | Shandong, Chian | A | 4 | 0 |
| Pt-10 | 2021 | Hebei, Chian | A | 3 | ； |
| Pt-11 | 2023 | Hebei, Chian | A | 3+ | 0 |
| Pt-12 | 2021 | Henan, Chian | A | 3+ | ； |
| Pt-13 | 2023 | Hebei, Chian | A | 4 | ； |
| Pt-14 | 2023 | Hebei, Chian | A | 4 | ； |
| Pt-15 | 2023 | Shandong, Chian | A | 3+ | ； |
| Pt-16 | 2023 | Shandong, Chian | A | 4 | ;+ |
| Pt-17 | 2023 | Hebei, Chian | A | 3 | 0 |
| Pt-18 | 2023 | Hebei, Chian | A | 3 | ； |
| Pt-19 | 2023 | Henan, Chian | A | 4 | ； |
| Pt-20 | 2023 | Hebei, Chian | A | 3 | 0 |
| Pt-21 | 2023 | Hebei, Chian | A | 4 | ； |

**Supplementary Table S2. Information of 168 *Pt* standard samples collected in 2024.**

| Line  No. | Isolate Codes | Collection area | Virulence  test | SNP genotyping |
| --- | --- | --- | --- | --- |
| 1 | HEB-101 | Hebei | V | **-** |
| 2 | HEB-102 | Hebei | V | **-** |
| 3 | HEB-103 | Hebei | A | **+** |
| 4 | HEB-104 | Hebei | A | **+** |
| 5 | HEB-105 | Hebei | V | **-** |
| 6 | HEB-106 | Hebei | A | **+** |
| 7 | HEB-107 | Hebei | V | **-** |
| 8 | HEB-108 | Hebei | V | **-** |
| 9 | HEB-109 | Hebei | V | **-** |
| 10 | HEB-110 | Hebei | A | **+** |
| 11 | HEB-111 | Hebei | A | **+** |
| 12 | HEB-112 | Hebei | A | **+** |
| 13 | HEB-113 | Hebei | V | **-** |
| 14 | HEB-114 | Hebei | A | **+** |
| 15 | HEB-115 | Hebei | V | **-** |
| 16 | HEB-116 | Hebei | A | **+** |
| 17 | HEB-117 | Hebei | A | **+** |
| 18 | HEB-118 | Hebei | V | **-** |
| 19 | HEB-119 | Hebei | A | **+** |
| 20 | HEB-120 | Hebei | A | **+** |
| 21 | HEB-121 | Hebei | V | **-** |
| 22 | HEB-122 | Hebei | A | **+** |
| 23 | HEB-123 | Hebei | V | **-** |
| 24 | HEB-124 | Hebei | V | **-** |
| 25 | HEB-125 | Hebei | A | **+** |
| 26 | HEB-126 | Hebei | V | **-** |
| 27 | HEB-127 | Hebei | A | **+** |
| 28 | HEB-128 | Hebei | V | **-** |
| 29 | HEB-129 | Hebei | A | **+** |
| 30 | HEB-130 | Hebei | A | **+** |
| 31 | HEB-131 | Hebei | A | **+** |
| 32 | HEB-132 | Hebei | A | **+** |
| 33 | SD-101 | Shandong | A | **+** |
| 34 | SD-102 | Shandong | A | **+** |
| 35 | SD-103 | Shandong | A | **+** |
| 36 | SD-104 | Shandong | A | **+** |
| 37 | SD-105 | Shandong | V | **-** |
| 38 | SD-106 | Shandong | V | **-** |
| 39 | SD-107 | Shandong | A | **+** |
| 40 | SD-108 | Shandong | A | **+** |
| 41 | SD-109 | Shandong | A | **+** |
| 42 | SD-110 | Shandong | V | **-** |
| 43 | SD-111 | Shandong | A | **+** |
| 44 | SD-112 | Shandong | V | **-** |
| 45 | SD-113 | Shandong | V | **-** |
| 46 | SD-114 | Shandong | A | **+** |
| 47 | SD-115 | Shandong | A | **+** |
| 48 | SD-116 | Shandong | V | **-** |
| 49 | SD-117 | Shandong | A | **+** |
| 50 | HEN-101 | Henan | A | **+** |
| 51 | HEN-102 | Henan | V | **-** |
| 52 | HEN-103 | Henan | A | **+** |
| 53 | HEN-104 | Henan | V | **-** |
| 54 | HEN-105 | Henan | V | **-** |
| 55 | HEN-106 | Henan | V | **-** |
| 56 | HEN-107 | Henan | V | **-** |
| 57 | HEN-108 | Henan | A | **+** |
| 58 | HEN-109 | Henan | V | **-** |
| 59 | HEN-110 | Henan | A | **+** |
| 60 | HEN-111 | Henan | A | **+** |
| 61 | HEN-112 | Henan | A | **+** |
| 62 | HEN-113 | Henan | V | **-** |
| 63 | HEN-114 | Henan | A | **+** |
| 64 | HEN-115 | Henan | A | **+** |
| 65 | HEN-116 | Henan | A | **+** |
| 66 | HEN-117 | Henan | A | **+** |
| 67 | HEN-118 | Henan | A | **+** |
| 68 | HEN-119 | Henan | V | **-** |
| 69 | HEN-120 | Henan | A | **+** |
| 70 | HEN-121 | Henan | A | **+** |
| 71 | HEN-122 | Henan | V | **-** |
| 72 | HEN-123 | Henan | A | **+** |
| 73 | HUB-101 | Hubei | A | **+** |
| 74 | HUB-102 | Hubei | A | **+** |
| 75 | HUB-103 | Hubei | A | **+** |
| 76 | HUB-104 | Hubei | A | **+** |
| 77 | HUB-105 | Hubei | V | **-** |
| 78 | HUB-106 | Hubei | V | **-** |
| 79 | HUB-107 | Hubei | A | **+** |
| 80 | HUB-108 | Hubei | V | **-** |
| 81 | HUB-109 | Hubei | A | **+** |
| 82 | HUB-110 | Hubei | V | **-** |
| 83 | HUB-111 | Hubei | V | **-** |
| 84 | ZJ-101 | Zhejiang | A | **+** |
| 85 | ZJ-102 | Zhejiang | V | **-** |
| 86 | ZJ-103 | Zhejiang | A | **+** |
| 87 | ZJ-104 | Zhejiang | V | **-** |
| 88 | ZJ-105 | Zhejiang | V | **-** |
| 89 | ZJ-106 | Zhejiang | A | **+** |
| 90 | ZJ-107 | Zhejiang | A | **+** |
| 91 | ZJ-108 | Zhejiang | V | **-** |
| 92 | ZJ-109 | Zhejiang | A | **+** |
| 93 | ZJ-110 | Zhejiang | A | **+** |
| 94 | ZJ-111 | Zhejiang | A | **+** |
| 95 | ZJ-112 | Zhejiang | V | **-** |
| 96 | ZJ-113 | Zhejiang | A | **+** |
| 97 | GS-101 | Gansu | V | **-** |
| 98 | GS-102 | Gansu | A | **+** |
| 99 | GS-103 | Gansu | A | **+** |
| 100 | GS-104 | Gansu | V | **-** |
| 101 | GS-105 | Gansu | V | **-** |
| 102 | GS-106 | Gansu | A | **+** |
| 103 | GS-107 | Gansu | A | **+** |
| 104 | GS-108 | Gansu | A | **+** |
| 105 | SC-101 | Sichuan | A | **+** |
| 106 | SC-102 | Sichuan | A | **+** |
| 107 | SC-103 | Sichuan | A | **+** |
| 108 | SC-104 | Sichuan | A | **+** |
| 109 | SC-105 | Sichuan | A | **+** |
| 110 | SC-106 | Sichuan | A | **+** |
| 111 | SC-107 | Sichuan | A | **+** |
| 112 | SC-108 | Sichuan | A | **+** |
| 113 | SC-109 | Sichuan | V | **-** |
| 114 | SC-110 | Sichuan | V | **-** |
| 115 | SC-111 | Sichuan | A | **+** |
| 116 | SC-112 | Sichuan | A | **+** |
| 117 | SC-113 | Sichuan | V | **-** |
| 118 | SC-114 | Sichuan | A | **+** |
| 119 | SC-115 | Sichuan | V | **-** |
| 120 | SX-101 | Shanxi | A | **+** |
| 121 | SX-102 | Shanxi | A | **+** |
| 122 | SX-103 | Shanxi | V | **-** |
| 123 | SX-104 | Shanxi | A | **+** |
| 124 | SX-105 | Shanxi | A | **+** |
| 125 | SX-106 | Shanxi | A | **+** |
| 126 | SX-107 | Shanxi | V | **-** |
| 127 | JS-101 | Jingsu | A | **+** |
| 128 | JS-102 | Jingsu | A | **+** |
| 129 | JS-103 | Jingsu | A | **+** |
| 130 | JS-104 | Jingsu | A | **+** |
| 131 | JS-105 | Jingsu | A | **+** |
| 132 | JS-106 | Jingsu | A | **+** |
| 133 | JS-107 | Jingsu | A | **+** |
| 134 | JS-108 | Jingsu | A | **+** |
| 135 | JS-109 | Jingsu | A | **+** |
| 136 | JS-110 | Jingsu | A | **+** |
| 137 | JS-111 | Jingsu | A | **+** |
| 138 | JS-112 | Jingsu | A | **+** |
| 139 | JS-113 | Jingsu | A | **+** |
| 140 | JS-114 | Jingsu | A | **+** |
| 141 | YN-101 | Yunnan | A | **+** |
| 142 | YN-102 | Yunnan | A | **+** |
| 143 | YN-103 | Yunnan | A | **+** |
| 144 | YN-104 | Yunnan | A | **+** |
| 145 | YN-105 | Yunnan | A | **+** |
| 146 | YN-106 | Yunnan | A | **+** |
| 147 | YN-107 | Yunnan | A | **+** |
| 148 | YN-108 | Yunnan | A | **+** |
| 149 | YN-109 | Yunnan | A | **+** |
| 150 | AH-101 | Anhui | A | **+** |
| 151 | AH-102 | Anhui | A | **+** |
| 152 | AH-103 | Anhui | A | **+** |
| 153 | AH-104 | Anhui | A | **+** |
| 154 | AH-105 | Anhui | A | **+** |
| 155 | AH-106 | Anhui | A | **+** |
| 156 | AH-107 | Anhui | A | **+** |
| 157 | AH-108 | Anhui | A | **+** |
| 158 | AH-109 | Anhui | A | **+** |
| 159 | AH-110 | Anhui | A | **+** |
| 160 | HLJ-101 | Heilongjiang | A | **+** |
| 161 | HLJ-102 | Heilongjiang | A | **+** |
| 162 | HLJ-103 | Heilongjiang | A | **+** |
| 163 | LN-101 | Liaoning | A | **+** |
| 164 | LN-102 | Liaoning | A | **+** |
| 165 | HAN-101 | Hainan | A | **+** |
| 166 | HAN-102 | Hainan | A | **+** |
| 167 | GZ-101 | Guizhou | A | **+** |
| 168 | GZ-102 | Guizhou | A | **+** |

Note: "+" indicates a visible band detected by PCR amplification using the molecular marker.

**Supplementary Table S3. List of designed primer sequences.**

| Primer name | Primer sequence（5’-3’） | Primer size（bp） |
| --- | --- | --- |
| *Lr15* Molecular Marker | CCTTCTACCTCGCATTTGAACT  GCCTGTTGGGAGATGGACA | 878 |
| M1 | ATGCACTGCCTCTTCTACGTC  GGCCCGCCGTAGCCGTAGAG | 275 |
| M2 | ATGCACTGCCTCTTCTACGTC  TTTCATCAACAGACCCGGC | 291 |
| M3 | CCCTTCGGCGCTGGCTCT  TTAAGCCGATGTGCTGGG | 96 |
| M4 | CCCTTCGGCGCTGGCTCT  TCAACAGACCCGGCCCGC | 49 |
| M5 | CCCTTCGGCGCTGGCTCT  TCTTTCATCAACAGACCCGGC | 56 |
| M6 | CCCTTCGGCGCTGGCTCT  GGCCCGCCGTAGCCGTAG | 38 |
| M7 | CTACGGCTACGGCGGGCC  TTAAGCCGATGTGCTGGG | 76 |
| M8 | GCCGGGTCTGTTGATGAA  TTAAGCCGATGTGCTGGG | 61 |
| M9 | GCCGGGTCTGTTGATGAA  GAGTTATACCAGCCCCCG | 44 |
| M10 | ATGAAAGATGCTCCGGGG  TTAAGCCGATGTGCTGGG | 48 |
| M11 | ATGGCTACGGCGGACTGC  TCAACAGACCCGGCCCGCC | 83 |
| M12 | ATATGGCTACGGCGGACTGC  CCCCGGAGCATCTTTCAT | 84 |
| M13 | ATGGCTACGGCGGACTGC  TTAAGCCGATGTGCTGGG | 113 |
| M14 | ATGGCTACGGCGGACTGC  GAGTTATACCAGCCCCC | 96 |
| M15 | CCCTTCGGCGCTGGCTCT  GAGTTATACCAGCCCCCG | 79 |
| M16 | CCCTTCGGCGCTGGCTCT  CCCCGGAGCATCTTTCAT | 65 |
| M17 | ATGCACTGCCTCTTCTACGTC  TTAAGCCGATGTGCTGGG | 333 |
| M18 | ATGCACTGCCTCTTCTACGTC  CCCCCGGAGCATCTTTCAT | 304 |
| M19 | GCCTTCGGCGCTGGCTCTCT  TTACGCCGATGTGCTGGA | 96 |
| M20 | TGGGCTGTTGTTGAAAGATG  TTACGCCGATGTGCTGGA | 60 |
| M21 | ATGCACTGCCTCTTCTACGTC  TGGGCTGTTGTTGAAAGATGC | 300 |
| M22 | ATGCACTGCCTCTTCTACGTC  GCATCTTTCAACAACAGCCCA | 295 |
| M23 | ATGCACTGCCTCTTCTACGT  CCCGCCGTAGCCGTAGA | 279 |
| M24 | ATGCACTGCCTCTTCTACG  CCAGTCCGCCGTAGCCATA | 238 |
| M25 | ATGCACTGCCTCTTCTACG  AAGTTATACCAGCCCCTGG | 316 |
| M26 | ATGCACTGCCTCTTCTACG  ACAACAGCCCACCGCC | 286 |
| M27 | GCCTTCGGCGCTGGCTCT  CCCCTGGAGCATCTTTCAA | 67 |
| M28 | TCTACGGCTACGGCGGT  TTACGCCGATGTGCTGGA | 77 |
| M29 | CTACGGCTACGGCGGTGGG  CCAGTCCGCCGTAGCCATA | 59 |
| M30 | ATGCACTGCCTCTTCTACGT  CATCTTTCAACAACAGCCCA | 295 |
| *β-actin* | GTTCTACAACGAGCTCCGTGTC  GACATACATTGCTGGGCAAC |  |

**Supplementary Table S4.** **PCR cycling program**

| Procedure | | Denaturation  94℃ | Annealing  59℃ | Extension  72℃ | 4℃ | Number of cycles |
| --- | --- | --- | --- | --- | --- | --- |
| 1 | 1min | |  |  |  | 1 |
| 2 | 30 s | | 1min | 2min |  | 35 |
| 3 |  | |  | 10min |  | 1 |
| 4 |  | |  |  | store |  |

| **Supplementary Table S5. Effects of different EMS mutagenesis concentrations on germination rate.** | | | |
| --- | --- | --- | --- |
| EMS concentration (%) | Mutagenized seeds | Germination (%) | Abnormal plants |
| 0 | 100 | 100 | 0 |
| 0.4 | 100 | 52 | 0 |
| 0.5 | 100 | 44 | 0 |
| 0.6 | 100 | 21 | 0 |
| 0.7 | 100 | 19 | 0 |
| 0.8 | 100 | 19 | 0 |

**Supplementary Table S6. Statistical table of V15 virulence frequency by province.**

| Province | Total Samples | Virulent Strains (V15) | Virulence Frequency (%) | χ² | P | Significance |
| --- | --- | --- | --- | --- | --- | --- |
| Hebei | 32 | 14 | 43.75 | 3.601 | 0.165 | ab |
| Shandong | 17 | 6 | 35.29 | 1.092 | 0.579 | c |
| Henan | 23 | 9 | 39.13 | 0.942 | 0.624 | bc |
| Hubei | 11 | 5 | 45.45 | 2.124 | 0.346 | a |
| Zhejiang | 13 | 5 | 38.46 | 2.219 | 0.330 | bc |
| Gansu | 8 | 3 | 37.50 | 2.224 | 0.329 | c |
| Sichuan | 15 | 4 | 26.67 | 0.651 | 0.722 | d |
| Shanxi | 7 | 2 | 28.57 | 0.470 | 0.791 | d |
| Jiangsu | 14 | 0 | 0.00 | - | - | e |
| Yunnan | 9 | 0 | 0.00 | - | - | e |
| Anhui | 10 | 0 | 0.00 | - | - | e |
| Heilongjiang | 3 | 0 | 0.00 | - | - | e |
| Hainan | 2 | 0 | 0.00 | - | - | e |
| Liaoning | 2 | 0 | 0.00 | - | - | e |
| Guizhou | 2 | 0 | 0.00 | - |  | e |
| χ² | - | - | 19.234 | - | - | - |
| P | - | - | 0.007 | - | - | - |

Virulence Frequency (%) = (Virulent Strains / Total Samples) × 100%; Lowercase letters represent “P < 0.05”; -: Not applicable (no variation in virulence frequency); χ² and P are from chi-square tests comparing the three sampling locations within each province; Provinces with zero virulent strains in all sampling locations were excluded from chi-square analysis
